# 664. Patient and Observer Scar Assessment Scales 2.0 and 3.0 Are Correlated During Burn Scar Evaluation

**DOI:** 10.1093/jbcr/irag033.530

**Published:** 2026-04-06

**Authors:** Alison R Ross, Melissa M McLawhorn, Mary A Bellon, Melodi Beynam, Angela B Golding, Cara M Delatore, Taryn E Travis, Jeffrey W Shupp, Shawn Tejiram, Jamie G Oh, Bonnie C Carney

**Affiliations:** The Burn Center at MedStar Washington Hospital Center, Washington, DC; MedStar Health, Washington, DC; The Burn Center at MedStar Washington Hospital Center, Washington, DC; The Burn Center at MedStar Washington Hospital Center, Washington, DC; The Burn Center at MedStar Washington Hospital Center, Washington, DC; The Burn Center at MedStar Washington Hospital Center, Washington, DC; The Burn Center at MedStar Washington Hospital Center, Washington, DC; MedStar Washington Hospital Center, Washington, DC; MedStar Washington Hospital Center, Washington, DC; MedStar Washington Hospital Center, Washington, DC; The Burn Center at MedStar Washington Hospital Center, Washington, DC

## Abstract

**Introduction:**

Commonly accepted methods of subjective scar evaluation include the Vancouver Scar Scale (VSS) and the Patient and Observer Scar Assessment Scale (POSAS). POSAS is often considered superior to the VSS because of its incorporation of a patient’s own assessment of their scar in addition to clinician assessment. POSAS 2.0 was launched in 2005 and based on patient and clinician feedback, an updated POSAS 3.0 was published in 2023. Additional symptom details were added and the numeric scale was changed to verbal descriptive responses to improve clarity.

Our center has used POSAS 2.0 to evaluate burn scar for over 10 years, but recognize the potential benefit of a transition to the 3.0 scale. This study sought to compare 2.0 and 3.0 scales to understand if they correlate with one another. It was hypothesized that 2.0 scores would correlate with 3.0 scores.

**Methods:**

Patients with burn scars (n = 15) were enrolled in a prospective study. Before treatment, they completed POSAS 2.0 and 3.0. Each patient and observer evaluated 2 scars (n = 30 scars). 2.0 is scaled using a numerical rating from 1-10. 3.0 is scaled using words/categories. For the 3.0 scale, the words were coded into numbers (e.g., not = 2, minimally = 4, etc.). The different symptom-specific questions were tested for correlation using Pearson’s R test between the two scale versions (e.g., color, overall, vascularity, etc.).

**Results:**

The majority of patients were male (n = 10, 67%), 7 patients identified as Black, and Fitzpatrick Skin Type (FST) spanned Type 2-5. The average age of patients was 46.7 years. Average age of the scar at time of evaluation was 11.5 months. POSAS 2.0 and 3.0 were strongly correlated (observer r = 0.92, patient r = 0.86, *p*<.0001) for the average scores. For the color-specific question, there was also strong correlation (observer r = 0.88, patient r = 0.80). Patients with FST 5 were even more strongly correlated for color (observer r = 0.91, patient r = 0.82). For both patient and observer symptom-specific questions, there was strong correlation (>r = 0.8) between the scale versions.

**Conclusions:**

POSAS 3.0 is a comparable measurement to 2.0 for subjective assessment of burn scar. In future work, results from 2.0 can be used as comparisons for future studies that may only utilize 3.0.

**Applicability of Research to Practice:**

POSAS has been used in the clinical setting as documentation of scar improvement after treatment. As providers move to 3.0, it is important to ensure the ability to make longitudinal comparisons of treatment effectiveness. This study shows that 3.0 can be compared to 2.0 without difficulty.

**Funding for the study:**

This work was funded in part by the Charles and Mary Latham Fund. This work was funded in part by an award from the National Center for Advancing Translational Science (NCATS/NIH).

